# Inhaler Technique and Adherence to Inhaled Medications among Patients with Acute Exacerbation of Chronic Obstructive Pulmonary Disease in Vietnam

**DOI:** 10.3390/ijerph16020185

**Published:** 2019-01-10

**Authors:** Chau Quy Ngo, Dung Minh Phan, Giap Van Vu, Phu Ngoc Dao, Phuong Thu Phan, Hanh Thi Chu, Long Hoang Nguyen, Giang Thu Vu, Giang Hai Ha, Tung Hoang Tran, Bach Xuan Tran, Carl A. Latkin, Cyrus S. H. Ho, Roger C. M. Ho

**Affiliations:** 1 Department of Internal Medicine, Hanoi Medical University, Hanoi 100000, Vietnam; ngoquychaubmh@gmail.com (C.Q.N.); phandungminh1996@gmail.com (D.M.P.); vuvangiap@hmu.edu.vn (G.V.V.); daongocphuhmu@gmail.com (P.N.D.); thuphuongdr@gmail.com (P.T.P.); 2 Respiratory Center, Bach Mai Hospital, Hanoi 100000, Vietnam; chuthihanhbmh@gmail.com; 3 Center of Excellence in Behavioral Medicine, Nguyen Tat Thanh University, Ho Chi Minh City 70000, Vietnam; longnh.ph@gmail.com (L.H.N.); pcmrhcm@nus.edu.sg (R.C.M.H.); 4 Center of Excellence in Evidence-Based Medicine, Nguyen Tat Thanh University, Ho Chi Minh City 70000, Vietnam; giang.coentt@gmai.com; 5 Institute for Global Health Innovations, Duy Tan University, Da Nang 55000, Vietnam; giang.ighi@gmail.com; 6 Department of Lower Limb Surgery, Vietnam-Germany Hospital, Hanoi 100000, Vietnam; tranhoangtungvdh@gmail.com; 7 Institute for Preventive Medicine and Public Health, Hanoi Medical University, Hanoi 100000, Vietnam; 8 Johns Hopkins Bloomberg School of Public Health, Baltimore, Maryland, MD 21205, USA; carl.latkin@jhu.edu; 9 Department of Psychological Medicine, National University Hospital, Singapore 119074, Singapore; cyrushosh@gmail.com; 10 Department of Psychological Medicine, Yong Loo Lin School of Medicine, National University of Singapore, Singapore 119077, Singapore

**Keywords:** COPD, inhaler technique, adherence, TAI, Vietnam

## Abstract

Sub-optimal chronic obstructive pulmonary disease (COPD) management has been found largely due to patients’ medication non-adherence and incorrect inhaler technique. This study aimed to examine inhaler use technique and medication adherence among Vietnamese COPD patients as well as potential associated factors. A cross-sectional study involving 70 COPD exacerbators was conducted. Inhaler technique and adherence were evaluated by the 10-item and 12-item Test of Adherence to Inhaler (TAI). Data on the history of COPD, home prescription of inhalers and duration of hospitalization were also collected. Generalized linear regression models were used to determine the associated factors with inhaler use and medication adherence. The results showed that the proportion of patients with good inhaler technique was 22.7% for metered-dose inhalers (MDI), 30.4% for dry powder inhalers (DPI) and 31.8% for soft-mist inhalers (SMI). Full exhalation was the most common mistake. The rates of non-compliance patterns were: “ignorant” (77.1%), “sporadic” (58.6%), and “deliberate” (55.7%). Worse dyspnea, greater health condition impairment, and an increased frequency of exacerbations and hospitalizations were found to be associated negatively with correct inhaler use and treatment adherence. Instructions to COPD patients about using inhalers should focus on correct inhaler technique and adherence even when feeling healthy.

## 1. Introduction

Chronic obstructive pulmonary disease (COPD) has been considered a significant global health issue, with increasing prevalence causing a substantial burden of disease for those infected and on society [1]. Furthermore, the growth of the prevalence and burden of COPD has been predicted to be on the rise, as a result of the hardly changing popularity of smoking, increasing air pollution and general aging of the world population [2]. According to recent projections, COPD will become the third leading cause of death and the seventh leading cause of disability-adjusted life years (DALYs) worldwide by 2030, exhibiting a major increase in terms of mortality and DALY-caused rankings in 1990, which were sixth and twelfth, respectively [3]. COPD-induced deaths have been particularly high in low and middle-income countries (LMICs), accounting for more than 90% of the total COPD fatalities worldwide [1], signaling that possibly more attention and effort should be placed on COPD prevention and control in these regions.

Despite becoming a rising global health threat, COPD has still largely been regarded as a preventable and treatable health problem [2]. Recommended strategies for COPD management commonly involve the use of inhaled medication [4]. It has been generally accepted that delivering medications via inhalation would allow for accurate and direct administration of medications to the intended area, while systemic absorption would be reduced, which in turn increases the possibilities of clinical benefit utilization and decreases the risk of potential side effects [5]. However, a number of studies have indicated that such COPD management strategies have not succeeded in providing optimal efficiency, with medication non-adherence and incorrect inhaler technique found in patients being cited as the main reasons [4,6,7]. Understanding the barriers to optimal inhaler use among patients as well as determining solutions to overcome these obstacles can be said to be essential to enhancing the effectiveness of COPD treatment.

Although the prevalence and severity of COPD in Vietnam has been reported in recent literature—the prevalence rate was found to be 6.7% in a study of COPD in 12 Asian countries [8], while another study indicated that over half of those infected experienced exacerbation episodes [9]. Despite this, there has been a lack of research looking into the use of inhalers among Vietnamese COPD patients. This study aimed to examine the current situation regarding inhaler use technique and adherence in a cohort of COPD patients in Vietnam as well as factors potentially influencing such use, with a hope to identify implications and improve medication effectiveness.

## 2. Methods

### 2.1. Study Design and Participants

A cross-sectional, observational study was conducted at the Respiratory Center of Bach Mai hospital, a tertiary hospital in Northern Vietnam. We recruited 70 COPD patients that were hospitalized due to acute exacerbations of COPD (AECOPD) from September 2018 to October 2018. The inclusion criteria were: (1) Patients admitted to the Respiratory Center due to AECOPD; (2) patients currently using at least one inhaler device at home for COPD management; and (3) patients able to be interviewed having agreed to participate in the study. Patients who had an acute state of respiratory failure or severe mental illnesses were excluded. A convenient sampling method was adopted. A total of 70 patients participated in the study.

### 2.2. Data Collection and Outcome Assessment

We collected patient data from medical records to determine the eligibility of participants. Information about history of COPD, prescription of inhalers at home, and the length of hospitalization was also collected. Face-to-face interviews were conducted after patients stabilized the acute condition. We asked patients to demonstrate how they used their inhalers at home and to answer the 12-item Test of Adherence to Inhaler (TAI).

#### 2.2.1. Inhaler Use Technique

Inhaler use technique was classified as poor and good according to the list of standards which were developed based on the 12-item TAI. We measured the use of metered-dose inhalers (MDI), soft-mist inhalers (SMI) and dry powder inhalers (DPI) among patients. Inhaler use technique was poor if the patients had any errors that might result in the loss of dose or reduction of drug delivery to the lung. Good inhaler use was defined as error-free. The items used to measure inhaler use are listed in Supplement S1.

#### 2.2.2. Medication Adherence

We used the Vietnamese version of the TAI to assess patients’ level of adherence (Supplement S2). The TAI is a questionnaire that assesses the adherence among patients with asthma or COPD [10]. The 10-item TAI consists of 10 questions. The scoring range for each question is from 1 (worst compliance) to 5 (best compliance), resulting in a total score from 10 (minimum) to 50 (maximum). Patients were classified based on the score as follows:

- 10-item scores equal or less than 45 (≤45): Poor adherence
- 10-item scores from 46 to 49 (46–49): Intermediate adherence
- 10-item scores of 50: Good adherence

Meanwhile, the 12-item TAI was used to explore non-adherence patterns. An additional two questions were used by the healthcare professionals, scoring from 1 (poor knowledge of the regimen and/or inhalation technique) to 2 (good knowledge). The types were categorized as below:

- Items 1–5 score less than 25: “sporadic non-compliance” (<25)
- Items 6–10 score less than 25: “deliberate non-compliance” (<25)
- Items 11–12 score less than 4: “ignorant” non-compliance” (<4)

#### 2.2.3. Assessment of Clinical Outcomes

Symptoms of COPD were also assessed using the Modified Medical Research Council scale of dyspnea (mMRC) grade ranging from 0–4 and the COPD Assessment Test (CAT) score ranging from 0–40. A higher CAT score means more severe symptoms.

### 2.3. Statistical Analysis

Data analysis was performed using SPSS software version 23.0 (SPSS Inc., Chicago, IL, USA) Descriptive statistics were adopted to examine patient characteristics. Generalized linear regression models, with binomial family and log link, were used to determine the associated factors with good/poor inhaler use and complete/not complete medication adherence. *p*-values less than 0.05 were considered statistically significant.

### 2.4. Ethical Approval

The purpose of the study was clearly explained to the participants and questionnaires were given to those who were willing to participate. The participants were informed that they had the right to withdraw at any time they wanted without any negative consequences to their current treatment. Patients’ information was kept secret. All information was encoded without revealing names and personal information. All participants provided written informed consents. The study protocol was approved by the Institutional Review Board of the Vietnam Respiratory Society (Code 03/QD-VNRS).

## 3. Results

Of the 70 participants, the mean age of patients was 68.6 (SD = 8.7) years old. The majority of patients were male (92.9%) and former smokers (82.9%). The mean number of pack-years was 31.1 ± 22.3 and the mean duration of COPD was 6.7 (SD = 7.5) years. According to GOLD 2017 classification, most patients were classified in group D (75.7%) (Table 1).

Table 2 shows that MDI users dominated the sample with 94.2%, followed by DPI and SMI users with 32.9% and 31.4%, respectively. Errors were more prevalent among MDI users compared to other groups. Full exhalation was the most common mistake at 72.8%, 69.6%, and 63.7% for MDI, DPI, and SMI, respectively. The proportion of patients with good inhaler technique in the MDI, DPI and SMI groups were 22.7%, 30.4% and 31.8%, respectively. Overall, 17 patients (24.3%) had good inhaler technique.

Table 3 reveals that 50% of the participants had poor adherence. The rates of non-compliance patterns for “Ignorant”, “sporadic”, and “deliberate” were 77.1%, 58.6%, and 55.7%, respectively.

The results of the multivariate regression models are shown in Table 4. People with a higher number of exacerbations in the last 12 months, higher CAT score and higher duration of hospitalization were less likely to have good inhaler technique. Meanwhile, those with a higher mMRC grade had a higher likelihood of having good inhaler technique (OR = 3.52; 95% CI = 1.22–10.12).

Regarding adherence, a higher CAT score was negatively associated with complete adherence (OR = 0.71; 95% CI = 0.58–0.87), while a higher duration of COPD was positively related to complete adherence (OR = 1.18; 95% CI = 1.02–1.36).

## 4. Discussion

Incorrect inhaler use technique and medication non-adherence were demonstrated to be associated with poor treatment outcomes among COPD patients. This study indicates that a large proportion of COPD patients use their inhalers incorrectly, of which most MDI users made errors in their use. Moreover, the current study reveals a significantly high rate of non-adherers in COPD patients. Incorrect inhaler use and poor adherence were found to be associated with high CAT scores, short durations of COPD, low mMRC grades, high durations of hospitalization and high numbers of exacerbations in the last 12 months.

In our cohort, a high percentage of patients making at least one error varied across the types of inhalers and steps, which is consistent with other studies [11,12]. Full exhalation is the leading error in all patients regardless of inhaler types, followed by inhalation and breath holding. These errors could be translated to mean that the patients were not fully aware of the technique that should be performed when using inhalers [13]. We also found that MDI users had more errors compared to other groups, which was similar to other previous findings. Batterink et al. found that patients using MDI experienced more errors than patients using other tools [14]. Al-Showair et al. reported that up to 60% of patients with COPD using MDI breathed too fast [15].

In this study, despite the importance of adherence in maximizing the therapeutic efficacy, optimal adherence was only observed in 30% of patients with AECOPD. This finding is significantly lower than previous studies in other clinical settings such as Australia (37–42%) [13,16], Hungary (58.2%) [17] and in seven Latin American countries (54.1%) [18]. Specifically, the prevalence of “ignorance type”, “erratic type”, and “deliberate type” of non-adherence were 77.1%, 60%, and 54.3% respectively, which is considerably higher than a study in Spain, which found that the prevalence of “ignorance”, “erratic”, and “deliberate” types were 31.2%, 47.8%, and 34.1%, correspondingly [19]. In terms of the “ignorance pattern”, most of the patients had poor knowledge of their own management of COPD, while for “erratic pattern” patients, we observed that the majority of patients stopped using their preventive inhaler due to “feeling healthy or the absence of severe symptoms”. Agh et al. also indicated that patients might be shown to stop treatment when no clinical symptoms appeared [17]. Regarding the “deliberate pattern” group, more than half of patients did not adhere to their preventive inhaler deliberately, mostly due to the financial burden of medication or traveling to a healthcare facility monthly to get medication.

Treatment adherence is a complex behavior that is affected by many factors [20]. Our results are consistent with previous studies showing that poor health outcomes (higher CAT scores, lower mMRC scores and more frequent exacerbations) and a high frequency of hospitalization were associated with inhaler mishandling and suboptimal adherence [13,16,20,21,22]. However, in the current study, we did not see an association between incorrect inhaler use technique and optimal adherence. Prior studies argued that treatment adherence and outcome were conversely influenced by inaccurate inhaler technique as well as multiple inhaler uses [20,23]. We supposed that a small sample size and a high level of homogeneity in our participants led to no significant association. Moreover, due to the complexity in predicting medication adherence in patients with COPD, the literature emphasizes that it is hard to fully identify the predictive factors within poor adherence practice [24], suggesting the involvement of multilevel factors regarding individual, family and societal perspectives in controlling treatment adherence.

Our findings suggest several implications. First, healthcare workers should provide clear instructions for patients and their families about the steps for inhaler use and underline important points that patients might forget or miss. Further, patients could perform the technique before the medical staff for correction. Second, health professionals should routinely check the inhaler usage of patients and counsel them appropriately and in a timely fashion. The literature shows that patients receiving inhalation instructions from healthcare workers have better technique than those who are self-studying [25]. Finally, further studies about the multilevel factors (individual, family and societal belief and perceived barriers) associated with medication adherence and inhaler use technique are warranted to fully support patients in managing and controlling COPD.

Limitations on this study included self-reported inhaler use technique and patient adherence, which might have led to recall bias as well as an over or under-estimation of medication utilization. In clinical practice, although self-reported measures are not more accurate than electronic monitoring, they provide a rapid and cheap assessment with acceptable results, playing a critical role in managing the disease. Selection bias due to the convenient sampling method might also have occurred. In addition, some clinical characteristics of patients such as spirometry results and bronchodilator responsiveness, which might be potentially associated with inhaler use technique and adherence, were not obtained. Moreover, the results might not reflect the pattern of non-adherence and inhaler use technique in other settings.

## 5. Conclusions

In conclusion, poor inhaler use and medication adherence were observed among patients with AECOPD in this study. Moreover, worse dyspnea, greater health condition impairment, and more frequent of exacerbations and hospitalizations were found to negatively affect correct inhaler use and treatment adherence. Instructions for COPD patients about using inhalers should focus on correct inhaler technique and adherence to preventive inhaled medication regimes even when feeling healthy.

## Figures and Tables

**Table 1:** Sociodemographic and chronic obstructive pulmonary disease (COPD)-related characteristics (*n* = 70).

Characteristics	*n*	%
Sex, Male	65	92.9
Smoking status		
Current smoker	7	10.0
Past smoker	58	82.9
Never smoked	5	7.1
Impact of symptoms (CAT score range)		
Low (<10)	13	18.6
Medium (10–20)	15	21.4
High (21–30)	33	47.1
Very high (>30)	9	12.9
mMRC grade		
Grade 0	2	2.9
Grade 1	11	15.7
Grade 2	11	15.7
Grade 3	34	48.6
Grade 4	12	17.1
GOLD 2017 classification		
A	3	4.3
B	6	8.6
C	8	11.4
D	53	75.7
Characteristics	Mean	SD
Age	68.6	8.7
Duration of COPD (years)	6.7	7.5
Number of packs per year	31.1	22.3
Number of exacerbations in the last 12 months	2.4	2.1
Length of hospitalization	9.7	4.7
CAT score	20.1	8.5

CAT: COPD Assessment Test; mMRC: Modified Medical Research Council; COPD: chronic obstructive pulmonary disease.

**Table 2:** Frequency of critical errors according to type of inhaler.

Characteristics	MDI		DPI		SMI	
	*n*	%	*n*	%	*n*	%
Total	66	94.2	23	32.9	22	31.4
Errors						
1. Prepare dose	24	36.4	1	4.4	0	0.0
2. Full exhalation	48	72.8	16	69.6	14	63.7
3. Inhalation	25	37.9	8	34.8	2	9.1
4. Breath-holding	23	34.9	6	26.1	6	27.3
5. Rinse mouth	20	30.3	2	8.7	-	-
Good inhaler technique	15	22.7	7	30.4	7	31.8

MDI: metered-dose inhalers; DPI: dry powder inhalers; SMI: soft-mist inhalers.

**Table 3:** Level of adherence and pattern of non-adherence.

TAI Interpretation		*n*	%
10-item score	Poor adherence (≤45)	35	50.0%
	Intermediate adherence (46–49)	14	20.0%
	Good adherence (=50)	21	30.0%
Item 1–5 score	Erratic pattern (<25)	42	60.0%
	Non-erratic (=25)	28	40.0%
Item 6–10 score	Deliberate pattern (<25)	38	54.3%
	Non-deliberate (=25)	32	45.7%
Item 11–12 score	Ignorance pattern (<4)	54	77.1%
	Non-ignorant (=4)	16	22.9%

TAI: Test of Adherence to Inhaler.

**Table 4:** Factor associated with inhaler technique and level of adherence.

Characteristics	Good Inhaler Technique		Complete Adherence	
	OR	95% CI	OR	95% CI
Age	1.03	0.89; 1.19	1.05	0.94; 1.17
CAT score	0.53 ***	0.40; 0.71	0.71 ***	0.58; 0.87
Duration of COPD	1.09	0.79; 1.52	1.18 **	1.02; 1.36
GOLD level	1.92	0.60; 6.11	0.79	0.30; 2.11
mMRC grade	3.52 **	1.22; 10.12	2.00	0.79; 5.03
Number of packs per year	0.96 *	0.91; 1.00	1.01	0.98; 1.04
Current smoker (Yes/No)	1.82	0.20; 16.66	0.14 *	0.02; 1.38
Duration of hospitalization	0.50 **	0.28; 0.88	0.93	0.76; 1.13
Number of exacerbations in the last 12 months	0.05 ***	0.01; 0.35	0.14 **	0.03; 0.70
Good inhaler technique (Yes/No)			0.21	0.02; 2.39

CAT: COPD Assessment Test; mMRC: Modified Medical Research Council; COPD: chronic obstructive pulmonary disease; OR: Odds ratio; CI: Confident Interval. *p* < 0.1; ** *p* < 0.05; *** *p* < 0.01.

## Supplementary Materials

The following are available online at http://www.mdpi.com/1660-4601/16/2/185/s1, Supplementary Materials S1: List of critical errors in inhalation technique, Supplementary Materials S2: 10-item and 12-item TAI questionnaire.
