# Supplementary material for: Inhaler Technique and Adherence to Inhaled Medications among Patients with Acute Exacerbation of Chronic Obstructive Pulmonary Disease in Vietnam
Source: Int J Environ Res Public Health. 2019 Jan 10;16(2):185. doi: 10.3390/ijerph16020185 (PMC6351932; doi:10.3390/ijerph16020185)
Supplement: Supplementary file 1 [file ijerph-16-00185-s001.pdf]

Supplement S1. List of critical errors in inhalation technique

Metered-dose inhaler (MDI)<br>Soft-mist inhaler (SMI)	Dry powder inhaler (DPI)
• Does not take off the cap<br>• Prepare the dose wrong/Does not shake the inhaler before use and between each puff<br>• Presses before inhaling/Press the MDI several times in a single inhalation<br>• Inhales too quickly or too forcefully<br>• Does not hold their breath after inhaling<br>• Coughs while inhaling or interruption of the inhalation (cold freon effect)<br>• Does not rinse the mouth after inhalation	• Does not take off the the cap<br>• Does not load the dose correctly<br>• Turn the device upside down after preparing the dose (before inhaling)<br>• Blows into the device before inhaling<br>• Cannot inhale forcefully<br>• Does not hold their breath after inhaling<br>• Does not rinse the mouth after inhalation

Supplement S2. 10-item and 12-item TAI questionnaire

Circle the response option that best describes your current situation					
**Self-report 10-item TAI. Measurement of adherence and degree of adherence**					
1. How many times did you forget to take your regular inhalers in the last 7 days?	**All**	**More than half**	**About half**	**Less than half**	**None**
	1	2	3	4	5
	**Always**	**Almost always**	**Sometimes**	**Almost never**	**Never**
2. You forget to take your inhalers:	1	2	3	4	5
3. When you are feeling well, you stop taking your inhalers	1	2	3	4	5
4. At the weekend or when you go on holiday, you stop taking your inhalers	1	2	3	4	5
5. When you are anxious or sad, you stop taking your inhalers	1	2	3	4	5
6. You stop taking your inhalers out of fear of potential side effects	1	2	3	4	5
7. You stop taking your inhalers because you believe that they are of little help in treating your disease:	1	2	3	4	5
8. You take fewer inhalations than prescribed by your doctor	1	2	3	4	5
9. You stop taking your inhalers because you believe that they interfere with your day-to-day or work life?	1	2	3	4	5
10. You stop taking your inhalers because you have trouble paying for them?	1	2	3	4	5
**The following two questions must be answered by the patient's health care professional in accordance with the data that appear in the patient's medical record (question 11) and after confirming their inhalation technique (question 12)**					
11. Does the patient know or remember the regimen (dose and frequency) that they were prescribed?	**No**		**Yes**		
	1		2		
12. The patient's device inhalation technique	Has critical errors		Has no critical errors or correct		
	1		2		
